# Yield of repeat gastric biopsies and *Helicobacter pylori* serological assessment in Lynch syndrome

**DOI:** 10.1016/j.ctarc.2025.101081

**Published:** 2025-12-18

**Authors:** Jessica Vadaketh, Jake Konigsberg, Omar Elghawy, Kevin Dinh, Linda Zhu, Julia Youngman, Michaela Dungan, Marya Pulaski, Jessica M. Long, Kole H. Buckley, Bryson W. Katona

**Affiliations:** a Department of Medicine, Hospital of the University of Pennsylvania, Philadelphia, PA, USA; b Division of Gastroenterology and Hepatology, Perelman School of Medicine, University of Pennsylvania, Philadelphia, PA, USA; c Boston Medical Center, Division of Gastroenterology, Boston, MA, USA; d Division of Hematology-Oncology, Department of Medicine, Penn Medicine, Philadelphia, PA, USA; e King Center for Lynch Syndrome, University of Pennsylvania, Philadelphia, PA, USA

**Keywords:** Lynch syndrome, Gastric cancer, Upper endoscopy, Gastric intestinal metaplasia, Helicobacter pylori, Surveillance

## Abstract

**Background:**

Upper gastrointestinal surveillance in Lynch syndrome (LS) remains an ongoing debate; some guidelines recommend upper endoscopy with non-targeted biopsies of the gastric antrum/body to detect *Helicobacter pylori* (*HP*) and/or gastric intestinal metaplasia (GIM). However, whether non-targeted gastric biopsies should be repeated on successive upper endoscopies remains uncertain. Therefore, we aimed to determine the yield of repeat non-targeted gastric antrum/body biopsies and assess the *HP* seropositivity of a LS cohort.

**Methods:**

Clinical and pathology data were collected retrospectively from LS carriers who underwent upper endoscopy with non-targeted gastric biopsies. Plasma samples from a LS biobank were tested for *HP* IgG positivity.

**Results:**

Amongst 683 LS carriers, there were 291 (43 %) with 1+, 145 (21 %) with 2+, and 45 (7 %) with 3+ upper endoscopies with non-targeted gastric antrum and body biopsies performed. The overall prevalence of GIM on those endoscopies was 8 % and the rate of *HP* was 3 %. Of individuals without GIM detected on the initial upper endoscopy, 4 % had GIM identified on their second, and 2 % had GIM identified on a third or greater upper endoscopy after having two prior upper endoscopies without GIM identified. There was no additional *HP* identified on subsequent endoscopies. Plasma *HP* IgG positivity amongst 257 LS carriers was 14 %.

**Conclusions:**

Amongst a LS cohort undergoing serial upper endoscopies, repeat gastric antrum/body biopsies yielded new cases of GIM, providing support for consideration of non-targeted gastric biopsies on all upper endoscopies performed in LS. Additionally, although endoscopic *HP* detection rates are low, *HP* exposure in LS is more common.

## Introduction

Lynch syndrome (LS) is a common autosomal dominant hereditary cancer predisposition syndrome that increases the risk of a myriad of different cancer types, most notably colorectal and endometrial cancer [1]. LS is caused by pathogenic germline variants (PGVs) in genes associated with the DNA mismatch repair (MMR) pathway including *MLH1, MSH2, MSH6*, and *PMS2*, as well as *EPCAM* deletions, which lead to silencing of *MSH2* [2]. Amongst digestive tract cancers, after colorectal cancer, the cancer with the next highest risk in LS is gastric cancer (GC) [1]. The risk of GC in the general population in the Western world is approximately 1 %, while the risk in LS carriers can be as high as 9 % [1,3]. In LS the GC risk varies based on the causative gene, with the highest risk observed in *MSH2* carriers and the lowest in *PMS2* carriers [1]. The risk of GC in LS increases with age and 5-year survival after a diagnosis of GC was reported to be 61 % [4,5]. Risk factors associated with increased GC risk in LS include male sex, older age, PGVs in *MLH1* or *MSH2*, ethnicity (non-White, with Korean Americans having the highest risk), and increasing number of first-degree relatives with GC [6,7].

The mechanism of gastric carcinogenesis in LS remains uncertain. Several surveillance studies have alluded to gastric carcinogenesis occurring rapidly in LS potentially via an accelerated carcinogenesis pathway, similar to development of colorectal cancer in LS [8,9]. In these studies, development of GC during surveillance has typically occurred within two years of a previous surveillance exam [10]. Some studies have alluded to atrophic gastritis being a potential driver of gastric carcinogenesis in LS, while other studies have not demonstrated similarly high rates of advanced atrophic gastritis in LS-associated GCs [11–13]. Additionally, environmental factors such as *Helicobacter pylori* (*HP*) infection may also play a role in LS-associated GC. *HP* increases GC risk through the Correa cascade, where chronic gastric inflammation can lead to the development of gastric intestinal metaplasia (GIM) and then GC [14,15]. However, recent work demonstrated that unlike carriers of a PGV in a homologous recombination-associated gene such as *BRCA1* or *BRCA2*, it does not appear that *HP* interacts with a LS PGV to synergistically increase GC risk [16]. Although, a prior study did not indentify *HP* in LS families with GC, further supporting that *HP* may not be a strong driver of gastric carcinogenesis in LS-associated GCs [17].

To date there remains no consensus within guidelines on GC surveillance in LS carriers [10,18]. The National Comprehensive Cancer Network (NCCN) currently recommends upper endoscopic surveillance in LS carriers with a *MLH1, MSH2/EPCAM*, or *MSH6* PGV starting at age 30–40 and repeating every 2–4 years, and a similar approach may be considered for *PMS2* carriers on an individualized basis [1]. Additionally, these guidelines recommend performing non-targeted biopsies of the gastric antrum and body on at least the initial upper endoscopy to detect *HP* and/or GIM [1]. Furthermore, for individuals not yet undergoing upper gastrointestinal (GI) surveillance, one-time noninvasive *HP* testing is recommended [1]. While there is no consensus about performing endoscopic surveillance, most guidelines are in agreement with *HP* screening for individuals with LS [10].

Previous work from our group assessed the rate of GIM and *HP* on consecutive patients with LS who underwent upper endoscopy where non-targeted biopsies were performed of the gastric antrum and body, regardless of its endoscopic appearance. Our data showed a 5.5 % rate of GIM and 3.6 % rate of *HP* in LS carriers [19]. As knowledge about *HP* and GIM in LS may affect future GC surveillance strategies, these data provide support for the NCCN recommendation for non-targeted gastric biopsies. However, whether non-targeted gastric biopsies should be performed on every surveillance upper endoscopy remains uncertain. Furthermore, although rates of active *HP* infection in LS may be low, the level of prior *HP* exposure in LS remains uncertain as does whether prior *HP* exposure impacts future GC risk.

Herein, we aimed to determine the utility of repeat non-targeted gastric antrum and body biopsies in LS carriers undergoing upper GI surveillance. Additionally, we aimed to assess the seroprevalence of *HP* antibodies as a marker of prior *HP* exposure in a United States-based LS cohort.

## Methods

### Study cohort

This is a retrospective study of all individuals with LS seen at the King Center for Lynch Syndrome (a tertiary care LS referral center) who underwent upper endoscopy with non-targeted gastric antrum and body biopsies. This study was approved by the University of Pennsylvania’s Institutional Review Board. Individuals included in the study had a confirmed diagnosis of LS. Additionally, these individuals had gastric biopsies performed as part of their routine care, including four jumbo forceps biopsies obtained from the gastric antrum as well as four jumbo forceps biopsies obtained from the gastric body. Biopsies underwent standard clinical pathology evaluation within the Penn Medicine Department of Pathology and Laboratory Medicine including routine H&E staining. Immunostaining for *HP* was performed at the discretion of the pathologist and regular use of other additional specialized stains was not performed. Per standard practice of the Penn Medicine Department of Pathology and Laboratory Medicine, GIM was not subclassified as complete or incomplete on final pathology reports. These procedures were performed between September 2018 and December 2024, and data on demographics, personal cancer history, family history of GC in a first- or second-degree relative, and upper endoscopy findings was collected and input into a secure REDCap database.

### HP antibody detection

*HP* IgG was detected utilizing plasma samples from an IRB-approved LS biobank maintained by Penn Medicine’s King Center for Lynch Syndrome. All biobank participants had signed informed consent and had a peripheral blood sample obtained and processed to isolate plasma, which was then stored at −80 °C until subsequent assays were performed. A commercially available ELISA kit (abcam; AB178645) that detects *HP* IgG was used to determine *HP* IgG positivity in 257 plasma samples, each from a distinct LS carrier. Per the manufacturer’s instructions, plasma samples from each carrier were diluted 1:100 with IgG Sample Diluent (supplied in kit) and run in triplicate, along with both positive and negative controls. Briefly, 100 μL of each diluted sample and controls were aliquoted into individual wells of a 96 well *HP* Coated Microplate (supplied in kit) and incubated for 1 h at 37 °C. Each well was then washed three times with 300 μL of 1X Washing Solution (supplied in kit). 100 μL of HRP conjugate (supplied in kit) was then added to each well and allowed to incubate for 30 min at room temperature in the dark. The wells were again washed three times with 300 μL of 1X Washing Solution followed by 100 μL of TMB Substrate Solution (supplied in kit) and allowed to incubate for 30 min at room temperature in the dark. Then, 100 μL of Stop Solution (supplied in kit) was dispensed into each well. Lastly, absorbance at 450 nm and a reference wavelength at 620 nm was measured using a microplate reader (Molecular Devices; SpectraMax M2). Mean absorbance readings were then plotted on a standard curve to calculate *HP* IgG concentration in IU/mL. Samples with an IgG concentration >20 IU/mL were considered *HP* IgG positive, while concentrations <15 IU/mL were considered *HP* IgG negative. Samples with IgG concentrations between 15–20 IU/mL were considered equivocal and were retested. If IgG concentrations remained equivocal upon retesting, they were considered *HP* IgG negative. Importantly, prior to use with these LS plasma samples, this ELISA kit was validated against a small cohort of known *HP* positive and *HP* negative samples (data not shown).

### Statistical analysis

All statistical tests were conducted using R v4.3.2. Continuous variables were described with medians and interquartile ranges while categorical variables were described with counts and proportions. Baseline characteristics were assessed via independent Wilcoxon rank-sum tests, Fisher’s exact tests, and 2-sided Pearson chi-square analysis, as appropriate. A Fisher’s exact test was used instead of a chi-squared test when the expected count for any value was <5. P-values <0.05 were considered statistically significant. To evaluate how long individuals remained free of GIM on subsequent upper endoscopies and in years from their index upper endoscopy, a Kaplan–Meier survival analysis was utilized. Odds ratios and 95 % confidence intervals were calculated using multivariate logistic regression. As many patients underwent more than one upper endoscopy, we performed additional analyses that evaluated each upper endoscopy separately while appropriately accounting for repeated procedures within the same individual. These per-upper endoscopy analyses used two complementary approaches: generalized estimating equations (GEE) and generalized linear mixed models (GLMM). The outcome was whether GIM was present on a specific upper endoscopy. Predictors included age (per 10 years), sex, race, germline variant (gene group; reference *MSH6*), and the interval since the prior upper endoscopy (reference = index upper endoscopy with non-targeted gastric biopsies). GEE models accounted for the correlation among upper endoscopies from the same patient by clustering at the patient level, producing population-average odds ratios (ORs) with 95 % confidence intervals (CIs). We also ran GLMM analysis (data not shown), which incorporated a patient-specific random intercept, allowing for individual differences in baseline GIM risk, and produced subject-specific ORs with 95 % CIs. Estimates from both approaches were directionally consistent and of similar magnitude to those from the primary multivariable logistic regression, supporting the robustness of our findings across different analyses and confirming that the results were not driven by patients with more upper endoscopies.

## Results

### Gastric biopsies on upper endoscopy

There were 683 individuals with LS in the King Center for Lynch Syndrome’s database. Of the 683, 291 (43 %) individuals had at least one upper endoscopy with non-targeted gastric antrum and body biopsies. Additionally, 145 (21 %) individuals had two or more and 45 (7 %) individuals had three or more upper endoscopies performed during which non-targeted gastric antrum and gastric body biopsies were collected. The median age of the LS cohort was 48 [IQR 38–61] (Table 1), and there were 196 (67 %) females with 252 (87 %) individuals identifying as White. The PGV stratification was 52 (18 %) *MLH1*, 79 (27 %) *MSH2*, 79 (27 %) *MSH6*, 74 (25 %) *PMS2*, and 7 (2 %) *EPCAM* carriers. Among the cohort, 155 (53 %) had a personal history of cancer and 50 (17 %) had a family history of GC.

The overall prevalence of GIM detected on all upper endoscopies performed with non-targeted antrum and body biopsies was 8 % (Table 2). The majority of GIM cases were found in the gastric antrum with 16 (5 %), 13 (9 %), and 4 (9 %) on the first, second, and third or greater upper endoscopy respectively compared to 4 (1 %), 2 (1 %), and 1 (2 %) with GIM on gastric body biopsies on the first, second, and third or greater upper endoscopy respectively (Table 2). There was only 1 (0.03 %) case where GIM was found only in the gastric body and not in the gastric antrum; this individual only had two upper endoscopies with both showing GIM in only the gastric body. The rate of *HP* identified on upper endoscopies with non-targeted biopsies was 3 %. Six of the 8 cases (75 %) of *HP* identified on the first upper endoscopy were found in both the gastric antrum and body, while 1 of 8 (12.5 %) was found only in the gastric antrum, and 1 of 8 (12.5 %) was found only in the gastric body. There were no statistically significant differences between characteristics of those with GIM/*HP* identified on biopsies compared to those without identified GIM/HP (Table 1).

**Fig. 1.**
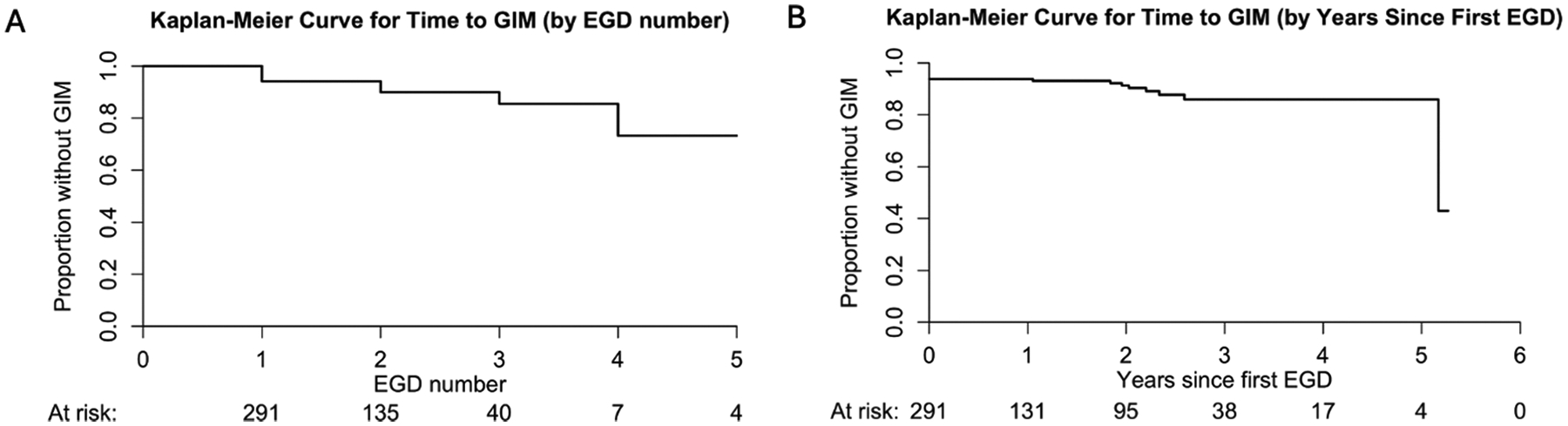
Kaplan–Meier analysis of time individuals with LS remained free of GIM across successive upper endoscopies (A), as well as over time since index upper endoscopy with non-targeted gastric biopsies (B). EGD = esophagogastroduodenoscopy (i.e. upper endoscopy).

There were 17 (6 %) LS carriers with GIM and 8 (3 %) with *HP* detected on the first endoscopy (Table 2). Subsequently, there were 6 (4 %) with newly identified GIM on the second upper endoscopy and 1 (2 %) with newly identified GIM on the third or greater upper endoscopy following two prior upper endoscopies with non-targeted biopsies of the gastric antrum and body without GIM and/or *HP*. In the multivariate logistic regression (Table 3), no demographic or genetic factors were statistically significantly associated with detection of GIM, although Asian ancestry showed a non-significant trend toward higher odds of having GIM and *PMS2* PGV carriers showed a nonsignificant trend toward lower odds of having GIM. In a GEE analysis, which accounted for repeated upper endoscopies within individuals, Asian ancestry showed higher odds of having GIM and *PMS2* with lower odds of having GIM. Additionally, in this analysis the results were directionally consistent with the logistic regression and were statistically significant (Table 3). Fig. 1A illustrates how GIM is incrementally detected in this population over successive upper endoscopies and Fig. 1B shows the increase in GIM over time after the index upper endoscopy with non-targeted gastric biopsies was performed.

Eleven individuals with GIM on their first upper endoscopy also underwent subsequent upper endoscopies; of these 8 (73 %) had persistent GIM identified on all subsequent upper endoscopies. There were no additional *HP* positive biopsies identified on subsequent upper endoscopies. There were no cases of GC diagnosed on upper endoscopy during the study period.

### HP IgG serology

**Fig. 2.**
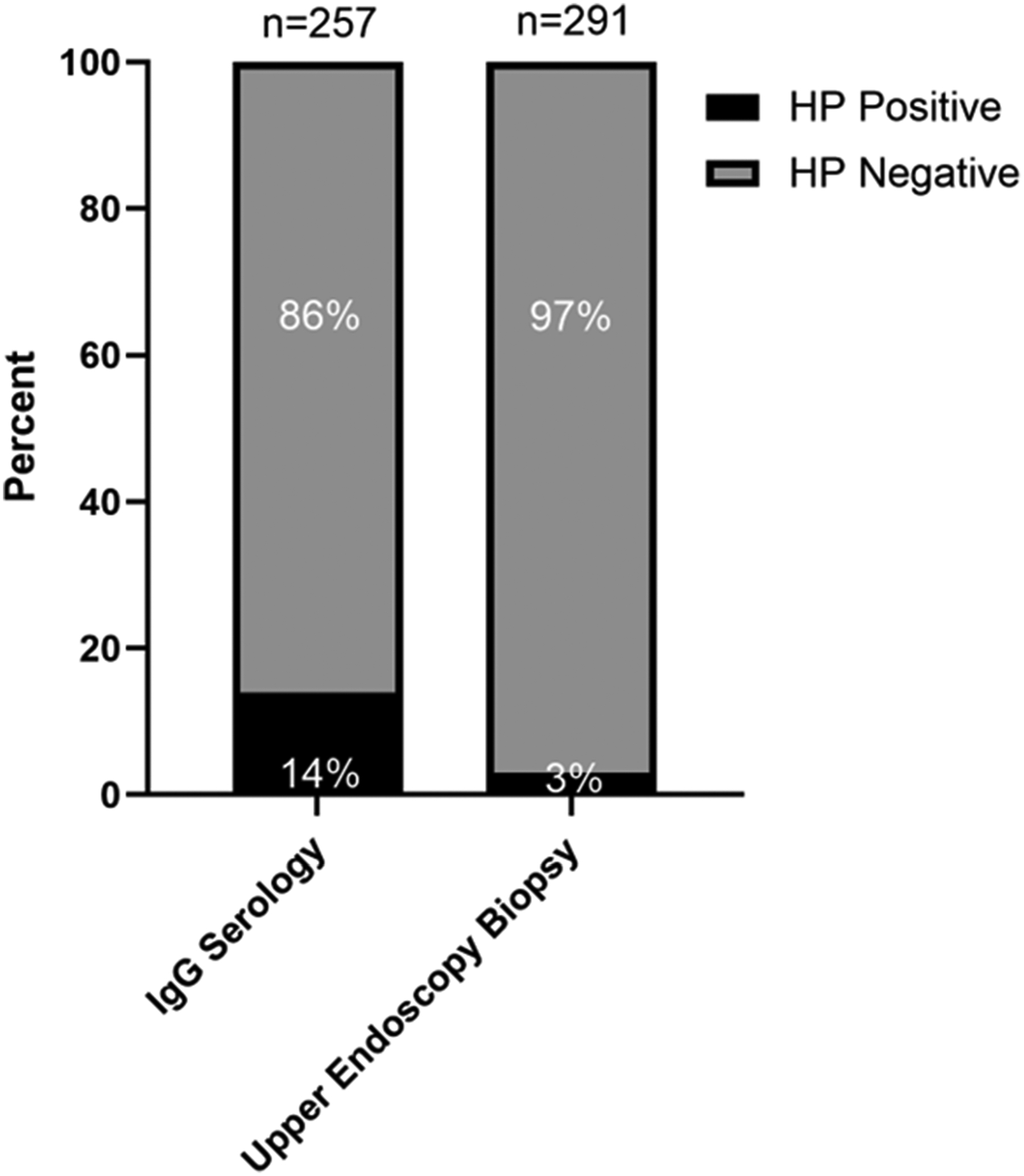
HP positivity on serology and gastric biopsy in LS cohorts.

There were 257 LS individuals with blood samples collected in the King Center for Lynch Syndrome biobank who underwent IgG serology testing for *HP*. This cohort differed from the cohort who underwent upper endoscopy with gastric antrum and body biopsies. The median age of the LS cohort that underwent IgG serology testing was 49 [IQR 38–62]. There were 171 (67 %) females and 231 (90 %) identified as White. The PGV stratification was 51 (20 %) *MLH1*, 72 (28 %) *MSH2*, 73 (28 %) *MSH6*, 54 (21 %) *PMS2*, and 7 (3 %) *EPCAM* carriers. There were 136 (53 %) individuals with a prior history of cancer and 35 (14 %) individuals with a family history of GC. *HP* IgG serology was positive in 36 (14 %) individuals consistent with prior *HP* exposure (Fig. 2). The characteristics of those with positive *HP* serology versus negative *HP* serology are shown in Table 4 with no statistical significance seen between the groups.

## Discussion

Upper GI surveillance in LS continues to be an important area for ongoing research given the limited outcome data available and the discordant recommendations amongst different guidelines. Furthermore, the field still lacks mechanistic understanding of LS-associated gastric carcinogenesis as well as understanding of important GC risk factors informing whether and how often a LS carrier should undergo upper GI surveillance. One area of great uncertainty is whether assessment for GIM and *HP* should be performed by non-targeted gastric biopsies on every surveillance upper endoscopy performed in LS. Herein we provide evidence that repeat non-targeted gastric biopsies in LS may have clinical utility during surveillance. Additionally, we demonstrate elevated rates of *HP* serologic positivity amongst a LS cohort, as compared to endoscopic *HP* detection rates, paving the way for future studies focusing on whether prior *HP* exposure affects future GC risk, to better inform upper GI surveillance strategies in LS.

Currently, there remains no consensus amongst professional organizations and their respective guidelines on GC surveillance in LS, and more specifically whether repeat non-targeted gastric biopsies should be performed on each subsequent surveillance upper endoscopy [10,18]. Therefore, it is first imperative that the prevalence of GC risk factors such as GIM and *HP* be defined in LS cohorts. We found that the prevalence of GIM in our LS cohort was 8 %, which is slightly higher than the estimated prevalence of GIM in the general US population (~4.8 %), thus indicating that individuals with LS do not appear to have a drastically higher rate of GIM compared to the average-risk population [20]. Furthermore, we found that the prevalence of *HP* was 3 % in our cohort, which is lower than would be expected in the general population, confirming that it is also unlikely that individuals with LS are at increased susceptibility to *HP* infection.

Importantly though, our data supports consideration of repeating non-targeted gastric biopsies on each upper endoscopy for LS surveillance, given the incremental identification of GIM over subsequent upper endoscopies. The rate of GIM identification on the first upper endoscopy was 6 % in our cohort, demonstrating that most GIM was identified on the initial set of non-targeted gastric biopsies performed. However, in contrast to gastric body biopsies, non-targeted gastric antrum biopsies still revealed newly identified GIM on subsequent upper endoscopies, with 4 % of individuals found to have GIM on a second upper endoscopy after the initial upper endoscopy did not have GIM identified. Furthermore, GIM was newly identified in one individual (2 %) after having two prior upper endoscopies with negative gastric biopsies. Detection of GIM did not depend on age, sex, race, or gene status, and since GIM was observed on subsequent upper endoscopies after initial gastric biopsies without GIM, the results support that non-targeted gastric biopsies may add value even after they are performed on the first upper endoscopy. The absence of a predictive patient profile makes a selective approach less reliable, strengthening the support for routine non-targeted gastric biopsies on all upper endoscopies in LS. In these cases of newly identified GIM, it is unlikely that the GIM acutely developed between surveillance upper endoscopies; given the often patchy nature of GIM across the gastric mucosa, in these cases it is more likely that GIM was present but not captured with initial sampling [21]. However, when considering repeated non-targeted gastric biopsies it is also important to consider other factors such as risk, cost, increased procedure time, and number needed to biopsy to detect new GIM. While repeated gastric biopsies are associated with a negligible increase in risk during the upper endoscopy, these biopsies do increase procedure time and may be associated with increased cost to the patient. Furthermore, the number of individuals with LS that need to be biopsied to detect a new case of GIM is high: 25 on the second upper endoscopy and 50 on subsequent upper endoscopies after the second upper endoscopy.

Another important question is whether both antrum and body biopsies need to be performed, or if antrum biopsies alone are sufficient. Prior work in a small LS cohort demonstrated that antrum biopsies may be sufficient for identifying all *HP* and GIM [19]. In this study, dedicated biopsies of the gastric body improved the diagnostic yield for both GIM and *HP* identification on initial upper endoscopy with gastric antrum and body biopsies. Interestingly, GIM was not newly identified in any non-targeted gastric body biopsies on subsequent upper endoscopies in our current study. This data supports that while gastric body biopsies may be important on the initial upper endoscopy, they may not have substantial clinical utility on subsequent upper endoscopies unless GIM and/or *HP* was identified on a prior upper endoscopy.

Whether GIM represents an independent risk factor for GC in LS remains to be determined. In a non-LS population, GIM is associated with a baseline 0.16 % annual risk of GC [22]. However, future long-term surveillance studies are needed in LS to determine if GIM is associated with increased GC risk in LS, and whether earlier identification of GIM can improve GC outcomes in LS. Furthermore, in our data we did not identify any patient- or family-specific factors that were associated with GIM and/or *HP* identification.

While active *HP* rates based on endoscopic biopsy were low in our study (3 %), 14 % of a LS cohort had positive *HP* IgG serology. Although previous research has shown a correlation between *HP* serology and *HP* positivity on endoscopic biopsy[23], this discrepancy could be associated with active *HP* infection that was not detected with endoscopic biopsy. However, in our cohort given that active HP was not newly detected on subsequent upper endoscopies, this argues against this possibility. Alternatively, this discrepancy could be related to higher rates of prior *HP* exposure amongst individuals with LS. The *HP* IgG seroprevalence in the United States is thought to be 31.5 % (stratified by age: 16.7 % for persons 20–29 and 56.9 % for those greater than or equal to 70 years old) [24]. However, what remains to be determined is whether prior *HP* exposure in LS portends any future increased GC risk. Such data will only be able to be assessed in large, longitudinal studies of LS upper GI surveillance where data is collected and available on *HP* serological status as well as gastric biopsy pathology.

Limitations to this study include that only a small number (*n* = 45) of LS carriers had 3 or more upper endoscopies, making the numbers for this subgroup small. Additionally, as previously mentioned, GIM and *HP* can be patchy in the gastric epithelium. Previous data showed that GIM and *HP* are not uniformly distributed, and thus non-targeted biopsies may miss some diagnoses of GIM and/or *HP* leading to underestimation of the prevalance [21,25]. Furthermore, most individuals with LS in our cohort were White females, therefore the results from this study might not be representative of other more diverse populations. Lastly, the cohort of individuals with gastric biopsy data did not exactly match the LS biobank cohort where *HP* serology testing was performed, therefore direct comparisons between serology and upper endoscopy biopsy findings could not be completed.

GC risk management in LS continues to be an important area for ongoing research. Herein we demonstrate that newly diagnosed GIM is identified on non-targeted gastric biopsies on subsequent upper endoscopies in LS; therefore, gastric biopsies should be considered on each surveillance upper endoscopy in LS. Additionally, *HP* serology rates are much higher than active infection rates in LS, indicating prevalent *HP* exposure in this cohort. Future studies developing algorithms to personalize upper GI surveillance intervals in LS that could potentially use GIM status and/or *HP* exposure, would be immensely beneficial for the field.

## Figures and Tables

**Table 1:** Characteristics of individuals with LS who underwent upper endoscopy with non-targeted gastric antrum and body biopsies with and without GIM and/or *HP*. P-values were computed using the Wilcoxon Rank-Sum test (age), Chi-squared test (sex, personal history), or Fisher’s exact test (all others).

Characteristic	Entire cohort (*N* = 291)	No GIM/*HP* detected (*N* = 267)	GIM and/or *HP* detected (*N* = 24)	P – value
**Age at first upper endoscopy, median (IQR), years**	48 (38–61)	48 (38–61)	48.5 (40–61)	0.66
**Female sex, No. (%)**	196 (67 %)	183 (69 %)	13 (54 %)	0.15
**Race, No. (%)**				0.31
White	252 (87 %)	232 (87 %)	20 (83 %)	
Black	10 (3 %)	10 (4 %)	0 (0 %)	
Asian	15 (5 %)	12 (4 %)	3 (13 %)	
Mixed/Other	14 (5 %)	13 (5 %)	1 (4 %)	
**LS gene with PGV, No. (%)**				0.16
*MLH1*	52 (18 %)	48 (18 %)	4 (17 %)	
*MSH2*	79 (27 %)	72 (27 %)	7 (29 %)	
*MSH6*	79 (27 %)	69 (26 %)	10 (42 %)	
*PMS2*	74 (25 %)	72 (27 %)	2 (8 %)	
*EPCAM*	7 (2 %)	6 (2 %)	1 (4 %)	
**Personal history of cancer, No. (%)**	155 (53 %)	139 (52 %)	16 (67 %)	0.17
**Familial history of GC, No. (%)**	50 (17 %)	47 (18 %)	3 (13 %)	0.78

**Table 2:** Incidence of GIM and HP in LS on successive upper endoscopies with non-targeted gastric antrum and body biopsies.

Gastric antrum and body biopsy findings, No. (%)	Any Upper Endoscopy (*n* = 291)	First Upper Endoscopy (n = 291)	Second Upper Endoscopy (n = 145)	3+ Upper Endoscopies (*n* = 45)
**Antrum + Body biopsies**				
GIM	24 (8 %)	17 (6 %)	14 (10 %)	4 (9 %)
New diagnosis of GIM	-	-	6 (4 %)	1 (2 %)
*HP*	8 (3 %)	8 (3 %)	0 (0 %)	0 (0 %)
**Antrum biopsies**				
GIM	23 (8 %)	16 (5 %)	13 (9 %)	4 (9 %)
New diagnosis of GIM	-	-	6 (4 %)	1 (2 %)
*HP*	7 (2 %)	7 (2 %)	0 (0 %)	0 (0 %)
**Body biopsies**				
GIM	4 (1 %)	4 (1 %)	2 (1 %)	1 (2 %)
New diagnosis of GIM	-	-	0 (0 %)	0 (0 %)
*HP*	7 (2 %)	7 (2 %)	0 (0 %)	0 (0 %)

**Table 3:** Multivariate logistic regression and GEE analysis to assess associations with detection of GIM.

Variable	OR	95 % CI	P-value
**Multivariate Logistic Regression Analysis**			
**Age (per 10y)**	1.13	(0.83, 1.55)	0.44
**Sex**			
Female (ref)	—	—	—
Male	1.62	(0.68, 3.89)	0.28
**Race**			
White (ref)	—	—	—
Asian	3.31	(0.81, 13.62)	0.10
Black	0	(0, inf)	1
Other	1.02	(0.12, 8.63)	0.98
**Gene**			
*MSH6* (ref)	—	—	—
*MLH1*	0.60	(0.17, 2.11)	0.43
*MSH2*	0.65	(0.23, 1.85)	0.41
*PMS2*	0.22	(0.05, 1.07)	0.06
*EPCAM*	1.20	(0.13, 11.37)	0.87
**Family history of gastric cancer**			
No (ref)	—	—	—
Yes	0.72	(0.20, 2.63)	0.62
Variable	Adjusted OR	95 % CI	P-value
**GEE Multivariate Analysis**			
**Age (per 10 years)**	1.33	(0.98, 1.81)	0.07
**Sex**			
Female (ref)	—	—	—
Male	1.74	(0.67, 4.55)	0.26
**Race**			
White (ref)	—	—	—
Asian	5.12	(1.228, 21.345)	0.03
Black	0	(0, 0)	0.000
Other	1.46	(0.16, 13.30)	0.74
**Gene**			
*MSH6* (ref)	—	—	—
*MLH1*	0.67	(0.17, 2.70)	0.57
*MSH2*	0.55	(0.17, 1.77)	0.31
*PMS2*	0.17	(0.03, 0.89)	0.04
*EPCAM*	2.32	(0.15, 36.03)	0.55
**Upper endoscopy number**			
First (ref)	—	—	—
Subsequent	1.77	(0.57, 5.52)	0.32

**Table 4:** Characteristics of individuals with LS who are seropositive or seronegative for HP IgG. Equivocal HP status after retesting was included as a negative result. P-values were computed using the Wilcoxon Rank-Sum test (age), Fisher’s exact test (race, gene), or Chi-squared test (others).

Characteristics	*HP* IgG positive (*N* = 36)	*HP* IgG negative (*n* = 221)	P-Value
**Age at collection, median (IQR), years**	59 (39.5–63.5)	49 (37–61)	0.12
**Female sex, No. (%)**	21 (58 %)	150 (68 %)	0.26
**Race, No. (%)**			0.69
White	33 (92 %)	198 (90 %)	
Black	1 (3 %)	6 (3 %)	
Asian	2 (6 %)	9 (4 %)	
Mixed/Other	0 (0 %)	8 (4 %)	
**LS gene with PGV, No. (%)**			0.28
*MLH1*	6 (17 %)	45 (20 %)	
*MSH2*	13 (36 %)	59 (27 %)	
*MSH6*	13 (36 %)	60 (27 %)	
*PMS2*	4 (11 %)	50 (23 %)	
*EPCAM*	0 (0 %)	7 (3 %)	
**Personal history of cancer, No. (%)**	22 (61 %)	114 (52 %)	0.34
F**amilial history of gastric cancer, No. (%)**	6 (17 %)	29 (13 %)	0.59
